# Leaders’ Future Orientation and Public Health Investment Intention: A Moderated Mediation Model of Self-Efficacy and Perceived Social Support

**DOI:** 10.3390/ijerph17186922

**Published:** 2020-09-22

**Authors:** Jianming Wang, Tsung Piao Chou, Chia-Pin Chen, Xiangzhi Bu

**Affiliations:** 1 School of Business Administration, Zhejiang University of Finance & Economics, Hangzhou 310018, China; sjwjm@zufe.edu.cn; 2 Department of Business Administration, National Chung Hsing University, Taichung 402, Taiwan; choutsungpiao@gmail.com (T.P.C.); cpchen@dragon.nchu.edu.tw (C.-P.C.); 3 Department of Business Administration, Business School, Shantou University, Shantou 515063, Guangdong, China

**Keywords:** leaders’ future orientation, public health policy, self-efficacy, perceived social support

## Abstract

Prior studies have investigated the issue of public health and health policy from economic, environmental, and healthcare perspectives. Research on public health from leaders’ perspective may also help to advance our knowledge about leaders’ perceptions, attitudes, and behavioral intentions toward public health management. Therefore, this study is based on social ideal theory, social cognitive theory, and social trust theory to investigate the influence of leaders’ future orientation on public health investment intention with the mediating role of leaders’ self-efficacy and the moderating role of perceived social support. Using a structural equation modeling with a sample data of 381 leaders of government agencies in Vietnam, empirical results indicate that leaders’ future orientation has a positive influence on public health investment intention. Furthermore, self-efficacy is found to have a positive mediating effect in the relationship between leaders’ future orientation and public health investment intention. In addition, perceived social support positively moderates the link between leaders’ future orientation and self-efficacy. Perceived social support also moderates the indirect effect of leaders’ future orientation on public health investment intention through self-efficacy. On one hand, this study contributes to theoretical research by clarifying the effects of leaders’ perceptions, and cognitive and behavioral intentions toward public health investment. Findings of this study may have implications for researchers who may have interest in studying the issue of public health management from leaders’ viewpoints. On the other hand, this study contributes to practitioners since understanding how leaders’ characteristics affect public health investment will enhance the quality of policy makers’ decision-making in improving public health for citizens and society.

## 1. Introduction

Healthcare can be provided by a public or a private provider. Public healthcare is usually provided by the government through national healthcare systems (e.g., national hospitals, clinics, and national healthcare organizations) whereas private healthcare is provided by a profit organization (e.g., private hospitals, clinics) [1,2]. Public health is one of the most important issues that every government has to care about [1]. Public health investment enhances human capital [2], increases human health [3], and brings well-being for citizens of a society [4]. An understanding of public health helps policy makers improve their strategy and management to enhance quality of public health for their society [1]. From citizens’ perspective, they often hope governments invest more in public health because they obtain more benefits from these investments [5]. For example, citizens benefit from programs such as disease protection, disability improvement, environmental protection, and other physical and mental health enhancement. However, from government leaders’ perspective, they have to consider the allocation of resources and the performance of their investment to public health programs [6]. This will affect their decision whether or not to invest more in public health programs [1].

Although prior studies have investigated the issue of public health and health policy, scholars often discuss this topic from economic, environmental, and healthcare perspectives [1,3,6,7]. For example, Bosworth, Cameron, and DeShazo [8] investigated residents’ willingness to pay for public health policies to treat illnesses. Tubbing, Harting, and Stronks [9] proposed an integrated concept of public health policy and used a sample data of 237 experts to measure the concept. Oliver et al. [10] determined the importance of collaboration and good relationships between researchers and policy makers in decisions of public health. Schönfeldt, Hall, and Pretorius [11] determined the relationship between food consumption and public health in South African context. Stassen, Gislason, and Leroy [12] examined the relationship between environmental disclosures and public health in European countries. Prior studies have provided rich evidence to the knowledge of public health management in current literature. However, research on public health management from leaders’ perspective is also worthy and necessary because several public health policies are related to government leaders’ decisions [6,7]. Due to the important role of public health system to citizens and society, it is often managed and controlled by government agencies [10,12]. The development of a public health system depends largely on leaders because they propose policy and decide to invest resource for public health system [2,5,10]. Furthermore, public health systems in emerging countries often operate under the centralized management of a government agency. The effectiveness and performance of a public health system in these countries is seriously influenced by government leaders [3]. Therefore, understanding public health management from leaders’ viewpoints in emerging countries will shed a new light to our knowledge of public health in current literature. In this study, we investigate the influence of leaders’ future orientation on public health investment intention with the mediating role of leaders’ self-efficacy and the moderating role of perceived social support.

To fill the research gap in current literature, this study enriches knowledge in public health research in three ways. First, leaders’ perceptions of time often play an important role in their decision-making [13]. Leaders who focus on long-term orientation often plan and implement policies that generate good outcomes [14]. These leaders also tend to care about goodwill and the well-being of society and of younger generations [15]. Unfortunately, the impact of leaders’ future orientation on their public health policy has been underdetermined in prior studies. This study is based on social ideal theory to explain the influence of leaders’ future orientation on their public health policy. Thus, this study contributes to current literature in which the relationship between leaders’ future orientation and their investment intention on public health is clarified in this study. Second, self-efficacy represents leaders’ perceptions of their ability to accomplish a certain task and obtain an objective [16,17]. According to social cognitive theory, a leader’s self-efficacy can make a difference in how they feel, think and act [18]. Therefore, leaders who focuses on long-term or short-term may have different personal action control in their ability and take a different action in their decision regarding to public health. In other words, leaders’ future orientation may influence their self-efficacy, which in turn affects their intention to invest in public health. By investigating the mediating role of self-efficacy, this study sheds a new light on our understanding of the mediating mechanism in the link between leaders’ future orientation and public health investment intention. Third, perceived social support refers to leaders’ perceptions that they will receive support from citizens, friends, and colleagues [19]. Based on social trust theory [20,21], this study argues for a moderating effect of perceived social support on the relationship between leaders’ future orientation and self-efficacy. This study also infers the moderating effect of perceived social support on the indirect effect of leaders’ future orientation on public health investment intention through self-efficacy. Thus, this study clarifies the moderating role of perceived social support of leaders which has not been determined in public health literature.

The structure of this study is organized as follows. The next section discusses concepts in the research model and develops relationships between variables. The third section describes the sample and data procedure. The fourth section presents the empirical results. The final section discusses research implications, limitations, and direction for future research.

## 2. Literature and Hypotheses

### 2.1. Public Health Policy

Public health policy is a broad concept which refers to “decisions, plans, and actions that are undertaken to achieve specific health care goals within a society” [22]. Public health is often a focus of attention for government, business, and individual citizens in a society [7]. A government agency is often responsible for the decision-making in public health policy, and leaders of these government agencies are qualified to do this job [5]. Several factors determine the investment in public policy. For example, leaders may consider the availability of resources, the importance of a specific public health program at a certain time, the influence of public health on society, and the willingness and demand of citizens for public health [1]. In the last decades, several social problems have called for more investment in public health, such as poverty, disability, malnutrition, environmental pollution, and various types of disease (Covid-19 pandemic) [22]. Unfortunately, public health generally receives significantly less government funding compared with medicine [23]. This study investigates leaders’ intention to invest more in public health programs from personal characteristics of individual leaders. This helps to understand public health issues from leaders’ perspective.

### 2.2. Leaders’ Future Orientation and Public Health Investment Intention

Future orientation reflects a person’s perceptions and hopes about the future. It is a type of motivation that prompts a person’s thinking about the future. It also presents the person’s value, expectancy, attitudes, and behavior toward the fulfillment of purposes [24]. Future orientation has been a focus of attention in various studies. For example, Zhu et al. [25] reported that future orientation plays an important role in people’s climate perception and decision-making. Thelken and Jong [14] found that future orientation has a positive impact on students’ attitudes toward sustainable entrepreneurship in European countries. Magee and Upenieks [26] suggested that future orientation differs between male and female in the United States, which affects their level of optimism about their future. Chekima et al. [27] stated that future orientation influences consumers’ attitudes and their consumption of food. Seginer and Mahajna [24] reported that students’ future orientation links perceived parenting and academic achievement. Chen and Kruger [28] found that future orientation mediates the relationship between perceived environmental cues and the likelihood of future success. These studies have provided rich evidence to demonstrate the influence of future orientation on peoples’ perceptions, attitudes, and behavior in their decision-making process.

Social ideal theory (SIT) states the idea that people often hope to live in an ideal society in which they can enjoy peace, justice, goodwill, and wellbeing [29,30]. In 1516, philosopher Thomas More proposed the concept of “Utopia”, which refers to an imaginary community or ideal society that possesses highly desirable or nearly perfect qualities for its citizens [31]. Ideal society has been discussed in several fields of study such as philosophy, political science, economics, sociology, and management [32]. The thought of ideal society may act as a motivation to guide people’s thinking, attitudes, and behavior. Specifically, political leaders often link their decision-making with the building up of an ideal society [31].

Although SIT has been widely used in political science and philosophy, its application in management and public health research is very limited. In this study, SIT can be used to explain the relationship between leaders’ future orientation and public health investment intention. According to SIT, leaders who hold a perception about an ideal society tend to care more about the future of a society [33]. That is, they tend to hold a vision that focuses on long-term benefits of a society [34]. These leaders are more likely to plan and implement policies that bring about the best for their society [35]. Public health is a core value of an ideal society. It represents the well-being that citizens can enjoy from governments’ policies [31]. For example, leaders who care about the future of a society often implement public health policies to attain the goals of sustainable development that, create more sustainable values for both citizens and governments [36]. The purpose of public health programs such as environmental protection, equality for disability, disease protection, nutrients for citizens, etc. is to promote greater health and well-being in a sustainable way, while strengthening integrated public health services and reducing inequalities [37]. Leaders who are oriented toward the future tend to plan and execute policies that attain the goals of public health because these public health programs contribute to the goodwill and wellbeing of a society [35]. In other words, leaders’ future orientation increase leaders’ willingness to invest more in public health. The following hypothesis is developed.

**Hypothesis** **1** **(H1).** *Leaders’ future orientation is positively related to public health investment intention*.

### 2.3. The Mediating Role of Self-Efficacy

Self-efficacy is a core construct of social cognitive theory [16,17]. It refers to “beliefs in one’s capabilities to mobilize the motivation, cognitive resources, and courses of action needed to meet given situational demands” [38] (p. 408). Bandura [17] stated that self-efficacy differs in different dimensions, including magnitude (a particular level of task difficulty), strength (the certainty of successfully performing a particular level of task difficulty), and generality (the extent to which magnitude and strength beliefs generalize across tasks and situations). Self-efficacy has been a focus of research in prior literature [39,40,41,42,43].

According to social cognitive theory [16,17], beliefs in self-efficacy lead to differences in how people think, feel, and act. A high level of self-efficacy enhances an individual’s confidence and quality of decision-making [18]. People with high self-efficacy tend to be motivated to choose to perform more challenging tasks [43]. Schwarzer et al. [18] suggested that a belief of self-efficacy can be obtained from an individual’s experience, verbal persuasion, emotion, cognitive process, and his/her characteristics. A leaders’ self-efficacy can be acquired from his/her cognitive and vision. For example, McCormick [44] stated that leaders who hold a high level of self-efficacy tend to make a high effective decision-making and they are more likely to be motivated by his/her orientation toward long-term strategy. Machida and Schaubroeck [45] also reported that leaders who focus on future development tend to hold high beliefs about their ability to overcome difficult tasks. Ng, Ang, and Chan [46] suggested that leaders who focus on future development tend to believe in their capability to obtain certain objective.

In the context of public health, leaders who are future-oriented often endeavor to think about how to create more values for future generations and generate more goodwill for society [47,48,49]. Given this belief in mind, leaders may be motivated to take action to overcome difficulties and face challenges. That is, focusing on the future development of a society, leaders may believe in their capability to accomplish certain task [47,50]. Furthermore, because public health is at the core of an advanced society in which people can enjoy well-being and goodwill [1], leaders who care about the future of their society and believe in their competences may care more about the improvement of public health for their citizens [51]. Consequently, future-orientation will motivate leaders and make them feel more confident about their capability to create more values for society [52]. These leaders may invest more to public health because they may believe that improvement of public health will benefit citizens and generate well-being for future generations. Thus, leaders’ future orientation will enhance leaders’ self-efficacy, which in turn increases leaders’ intention to invest more in public health. The following hypothesis is developed.

**Hypothesis** **2** **(H2).** *Self-efficacy positively mediates the relationship between leaders’ future orientation and public health investment intention*.

### 2.4. The Moderating Role of Perceived Social Support

Social trust theory refers to “an individual’s beliefs about the general trustworthiness of others and it is part of a person’s worldview regarding the benevolence of other human beings” [53] (p. 149). Social trust influences and shapes a person’s perceptions, attitudes, and behavior toward the social world. In other words, social trust shapes a person’s worldview, which guides his/her thinking, feeling and action [54]. Social trust has been reported to affect politicians’ decision-making. For example, leaders who hold a high level of social trust tend to engage in policies that benefit cultural minorities and immigrants [55]. Social trusting leaders also support internationalist foreign policies and favor cooperative foreign policies [56]. Furthermore, social trust also increases political leaders’ level of support for free trade, foreign aid, and participation in international institutions [57]. These studies have provided rich evidence on the influence of social trust on leaders’ perceptions, attitudes, and behaviors in their decision-making.

Perceived social support is defined as “the support received or interpersonal interactions (with relatives, friends, neighbors and members of social organizations) aimed at giving and receiving some kind of spiritual, emotional, instrumental or informational aid” [58] (p. 211). Perceived social support is an important concept in social trust theory [53,55]. When individuals perceive a high level of social support, they hold a high level of trust in society, which motivates them toward positive behavior (e.g., these people are more integrated and active in their communities and engage in pro-social behavior) [59]. Perceived social support is believed to act as a motivation to increase a person’s perceptions, attitudes, and behavior toward a positive outcome [19].

In the context of public health and health policy, the level of perceived social support may affect the relationship between leaders’ future orientation and their self-efficacy. According to social trust theory [53,60], when a leader holds a high perception of social support, he or she may trust the society and human beings. That is, leaders may feel optimistic and confident because they received supports from their citizens, colleagues, and friends [58]. In other words, given the perceptions of high level of social support from citizens and colleagues, leaders who care about the future and development of a society will believe that they have the ability and competences to contribute to society [57]. By contrast, when leaders do not receive support from their citizens and colleagues, they are less likely to care about the development and future of their society. These leaders may also be conservative and do not want to contribute to their society and help their citizens [59]. Thus, when leaders perceive a high or a low level of social support, they may have low or high motivation to focus on the long-term or short-term development of their society, and they may be also optimistic or pessimistic about their ability to contribute to society. Based on social trust theory and these arguments, it is expected that leaders’ perceptions of social support will have a moderating effect on the link between leaders’ future orientation and self-efficacy. The following hypothesis is developed.

**Hypothesis** **3** **(H3).** *Perceived social support positively moderates the relationship between leaders’ future orientation and self-efficacy such that the relationship is stronger when perceived social support is high and weaker when perceived social support is low*.

**Figure 1:**
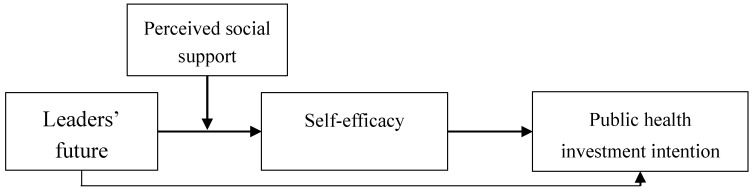
Research model.

This study proposes a research model as presented in Figure 1. It is hypothesized that self-efficacy mediates the relationship between leaders’ future orientation and public health investment intention (H2), and perceived social support moderates the relationship between leaders’ future orientation and self-efficacy (H3). If these hypotheses are correct, it is reasonable to infer that perceived social support moderates the indirect effect of leaders’ future orientation on public health investment intention through self-efficacy. Thus, the following hypothesis is developed.

**Hypothesis** **4** **(H4).** *Perceived social support positively moderates the indirect effect of leaders’ future orientation on public health investment intention through self-efficacy such that the indirect effect is stronger when perceived social support is high and weaker when perceived social support is low*.

## 3. Methods

### 3.1. Measures

This study used a five-point Likert scale to measure all items of the questionnaire. The scale was assigned from 1 (strongly disagree) to 5 (strongly agree). All items of the constructs were adopted from prior studies, which have provided evidence of reliability and validity for these measures. All items and constructs are shown in Table 1.

### 3.2. Sample and Data Collection

The questionnaire was designed with the assistance of four language translators. This study used a forward–backward translation method. The initial English items were translated to Vietnamese and back from Vietnamese to English. The final questionnaire was delivered to 6 doctorate students to help clarify and ensure the meaning of each item.

According to WHO’s report, the healthcare system in Vietnam is a mix of public and private systems. The public sector dominates the whole country’s healthcare system and is organized under administrative hierarchy. Government agencies have the authority and responsibility for every main policy of the public healthcare system. For example, leaders of government agencies decide to invest resources, human and other, on the public healthcare system. Most of major hospitals and healthcare organizations are located in Hanoi and Ho Chi Minh City, which are the two largest and most developed cities in Vietnam. According to Vietnam’s Ministry of Health, public hospitals in Hanoi and Ho Chi Minh City include 47 central hospitals, which are the highest level of the healthcare system and account for nearly 95% of all major hospitals in Vietnam. Furthermore, there are many other public hospitals at the provincial and district levels. However, these hospitals are small and a secondary level of public healthcare system in Vietnam. Due to Hanoi and Ho Chi Minh City being the two most important cities in the healthcare system in Vietnam, we selected Hanoi and Ho Chi Minh City as our research targets.

We obtained a sample list of leaders in different districts and counties in Hanoi and Ho Chi Minh City, and we randomly selected 500 leaders from this list and contacted them by telephone. We used a paper-based questionnaire and asked the respondents to complete the questionnaire when we visited them face-to-face. These leaders are presidents, vice presidents, secretaries, and vice secretaries of different districts and counties of a city. After 3 months of surveying, from June to September 2019, a total of 400 leaders agreed to participate in the survey and completed the questionnaire. A final sample of 381 questionnaires were valid with a response rate of 95.25%, and 19 questionnaires were invalid with missing data. The basic information of respondents is shown in Table 2.

### 3.3. Analysis Methods

To analyze empirical data, we adopted different statistical methods in this study. First, we used SPSS statistical software version 18 to screen and analyze descriptive statistics and the reliability of the measures. Second, we used Partial Least Square Structural Equation Model (PLS-SEM) to perform confirmatory factor analysis, check the validity of the measures, and test the hypotheses. Specifically, we adopted PLS-SEM to perform a confirmatory factor analysis (CFA) to test the fit between sample data and the hypothesized model. Based on results of this CFA model, we computed values for testing the reliability and validity of the measures. Furthermore, we also used PLS-SEM to teste all direct, mediating, and moderating effects in a single model.

To avoid potential impact of respondents’ characteristics on the results of hypothesis testing, we controlled for the impact of gender, age, marital status, income, education, and tenure in the analysis.

### 3.4. Ethical Consideration

To consider research that is involved with human activities, we clearly explained the purpose of our study to all respondents. Furthermore, to ensure the privacy and safety of the respondents, we used an anonymous questionnaire to protect the private information of respondents. After obtaining their voluntary participation, we asked them to sign a consent form. Because this study collected data from leaders in different government agencies, ethical consideration has been carefully checked and agreed by these leaders.

## 4. Results

### 4.1. Descriptive Statistics

The results of means, standard deviations, and correlation coefficients between variables are presented in Table 3. It is indicated that leaders’ future orientation was positively related to self-efficacy (r = 0.47, *p* < 0.01) and public health invention intention (r = 0.47, *p* < 0.01). Furthermore, self-efficacy was positively related to public health investment (r = 0.48, *p* < 0.01). In addition, perceived social support was positively related to self-efficacy (r = 0.40, *p* < 0.01).

### 4.2. Confirmatory Factor Analysis

Results of a confirmatory factor analysis (CFA) show a good fit between sample data and the hypothesized research model in this study. Specifically, the value of Chi-square/degree of freedom = 403.026/158 = 2.551, which was less than the cutoff value of 3; the values of goodness of fit index (GFI) = 0.91, comparative fit index (CFI) = 0.95, and Tucker–Lewis index (TLI) = 0.94 all exceeded the cutoff value of 0.90; root mean square error of approximation (RMSEA) = 0.06, which was less than the cutoff value of 0.08 [63].

### 4.3. Reliability and Validity

Reliability of the measures was tested using Cronbach’ α. Results in Table 4 show that Cronbach’s α of all variables were 0.92 (leaders’ future orientation), 0.91 (self-efficacy), 0.83 (perceived social support), and 0.87 (public health investment intention). These values exceeded the threshold value of 0.60 [63], which indicates a good reliability of the measures.

We followed Hair et al. [64] and used composite reliability (CR), average variance extracted (AVE), and square roots of AVE to test the validity of the measures in this study. Accordingly, convergent validity is supported if CR is greater than 0.70 and AVE exceeds 0.50. Results in Table 4 show that all CR values and AVE values satisfied this requirement. Thus, convergent validity is good for the measures in this study. Furthermore, discriminant validity is satisfactory if the values of square roots of AVE are all greater than correlation coefficients. Results in Table 3 show that all values of square roots of AVE exceeded all correlation coefficients. Therefore, discriminant validity is supported by the measures in this study.

### 4.4. Common Method Bias

This study also performed Harman’s one factor test. That is an exploratory factor analysis with unrotated solution. Results show a solution of four factors with 68.36% of variance, and the first factor accounted for only 27.43% of variance. Thus, according to Podsakoff et al. [65], neither only one factor emerges nor the first factor accounts for more than 50% of the variance; common method bias is not a serious problem. This result was confirmed with a one-factor model of CFA. Results indicate a poor model fit of this one-factor model: Chi-square/degree of freedom = 1728.964/170 = 10.17, GFI = 0.64, CFI = 0.69, TLI = 0.66, RMSEA = 0.16. Therefore, common method bias may not seriously affect the results of hypothesis testing.

### 4.5. Hypothesis Testing

**Figure 2:**
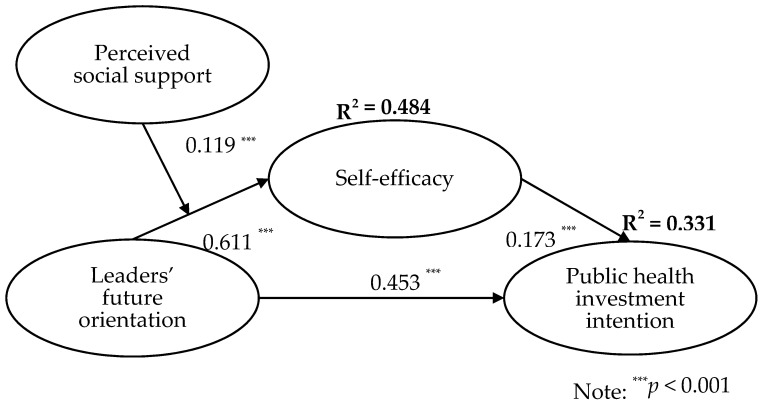
Hypothesis testing results.

This study used PLS-SEM to test all hypotheses in a single model. Results in Figure 2 show that leaders’ future orientation was positively associated with public health investment intention (β = 0.453, *p* < 0.001). This result supports hypothesis H1.

Furthermore, leaders’ future orientation was positively associated with self-efficacy (β = 0.611, *p* < 0.001), which in turn was positively related to public health investment intention (β = 0.173, *p* < 0.001). Results of a bootstrap analysis [66] with 1000 samples indicate that the indirect effect of leaders’ future orientation on public health investment intention through self-efficacy was statistically significant (β = 0.108, *p* < 0.001, 95% CI = (0.038, 0.234)). Thus, hypothesis H2 was supported.

In addition, results of PLS-SEM show that perceived social support positively moderated the relationship between leaders’ future orientation and self-efficacy (β = 0.119, *p* < 0.001). That is, the relationship between leaders’ future orientation and self-efficacy was stronger when perceived social support was high and weaker when perceived social support was low. Thus, hypothesis H3 is supported. Moreover, we followed Edwards and Lambert’s [67] procedure and tested the moderated mediation hypothesis. Results show that the indirect effect of leaders’ future orientation on public health investment intention was stronger for high social support group (β = 0.226, *p* < 0.001) and weaker for low social support group (β = 0.106, *p* < 0.001). This indirect effect was also significantly different between high and low social support groups (△β = 0.120, *p* < 0.01). Thus, hypothesis H4 was supported.

## 5. Discussion and Implications

This study investigates the influence of leaders’ future orientation on public health investment intention with the mediating role of leaders’ self-efficacy and the moderating role of perceived social support. Results of this study reveal several interesting findings. Leaders’ future orientation has a positive influence on public health investment intention. Furthermore, self-efficacy is found to have a positive mediating effect in the relationship between leaders’ future orientation and public health investment intention. In addition, perceived social support positively moderates the link between leaders’ future orientation and self-efficacy. Perceived social support also moderates the indirect effect of leaders’ future orientation on public health investment intention through self-efficacy. Findings of this study provide implications for both researchers and policy makers.

### 5.1. Theoretical Implications

First, prior studies have looked at public health from various perspectives including economic, environmental, and healthcare management [1,3,6,7]. Rowitz [68], Bradd, Travaglia, and Hayen [69], and Smith et al. [70] conducted a systematic review of leadership in public health literature. The authors pointed out the important role of leaders in public health policy. Several important characteristics of leaders influence public health decisions, including leaders’ personality, leadership style, goal alignment, communication, team building, and others. Unfortunately, few studies have determined the impact of leaders’ future orientation on public health decisions. The roles of self-efficacy of leaders and their perceptions of support from society are also underdetermined in prior literature. This study, based on social ideal theory, investigates the direct impact of leaders’ future orientation on their intention to invest in public health. Findings indicate that leaders’ future orientation enhances their intention to invest more in public health. That is leaders who focus on long-term orientation and care about the future of their society tend to plan and implement policies that bring the best for their society [35]. This will motivate leaders to invest in public health to improve the well-being of their citizens [31]. Thus, this study extends social ideal theory and provides initial evidence from the leadership’s perspective on the link between leaders’ future orientation and public health investment intention.

Second, due to the limited studies on public health from leaders’ perspective, the mediating effect of leaders’ self-efficacy in the relationship between leaders’ future orientation and public health investment intention has been underdetermined in current literature. Drawn from social cognitive theory, this study found the mediating role of leaders’ self-efficacy. This finding implies that leaders who are future-oriented often endeavor to think about how to create more values for future generations and generate more goodwill for society [47,48,49]. These leaders believe in their ability and competences to improve the quality of their society [47]. Consequently, these leaders tend to invest more in public health to bring well-being and goodwill for their citizens [51]. This study extends social cognitive theory by explaining the role of leaders’ self-efficacy to the issue of public health. Findings of this study also clarify the mediating mechanism of leaders’ self-efficacy into the link between leaders’ future orientation and public health investment intention. This has not been investigated in current public health literature.

Third, perceived social support also plays an important role in leaders’ decision-making. It indicates whether a leader’s policy is supported by citizens. Unfortunately, the role of perceived social support has been largely ignored in current public health literature. This study was based on social trust theory in order to demonstrate the moderating role of perceived social support in the link between leaders’ future orientation and self-efficacy and in the indirect effect of leaders’ future orientation on public health investment intention through self-efficacy. Thus, this study sheds a new light on the moderating mechanism of perceived social support and integrates social trust theory in the issue of public health in current literature. This finding may provide implications for researchers who may be interested in studying social trust and social support in leaders’ decision-making process.

Finally, this study tests the hypotheses by using a sample data of leaders of government agencies in Vietnam. Because Vietnam is an emerging economy which focuses mainly on economic development. Although public health is very important, it has not been invested as the first priority in government policy. Findings of this study provide initial evidence for researchers who may be interested in studying public health policy in emerging economies like Vietnam.

### 5.2. Practical Implications

This study also provides implications for government policy makers and individual citizens. It is suggested that policy makers should engage in a long-term strategy and policy which focus on the future development of society. Results demonstrated that leaders who focus on long-term orientation may invest more in public health. This result implies that policy makers should integrate public heath as a core and long-term strategy to develop a sustainable society. Furthermore, because self-efficacy represents leaders’ ability and competency to overcome difficulty and accomplish certain tasks, it is an important psychological factor that affects leaders’ decision making. Self-efficacy is found to have an influence on leaders’ intention to invest in public health in this study. Thus, it is suggested that leaders who engage in public health policy should understand the effect of self-efficacy. They may believe in their ability and competency to engage in policies that improve public health for their citizens and society. In addition, social support is very important for leaders to make and implement policy. If citizens oppose their leaders’ policy, the policy may face difficulties and not obtain final objectives. Therefore, it is suggested that leaders should understand and perceive whether their policies are supported by society and citizens. By contrast, based on the findings in this study, it is suggested that citizens should provide strong support for leaders who are future-oriented because these leaders tend to invest more in public health to bring more goodwill for society.

## 6. Conclusions

This study begins from leaders’ perspective to determine the issue of public health. Specifically, this study enriches knowledge about the effect of leaders’ psychological factors on their decisions about public health investment. Specifically, this study has clarified the direct impact of leaders’ future orientation on public health investment intention. Furthermore, this study also provided empirical evidence on the mediating mechanism of leaders’ self-efficacy and the moderating mechanism of perceived social support. The research model in this study helped to advance our knowledge about public health from leaders’ viewpoint.

This study has several limitations that need to be addressed in future research. Cross sectional data may affect the results of hypothesis testing because causal relationships between variables may be influenced by such data. Future research should collect data at different points in time (longitudinal data) to validate the relationship between variables in our research model. Furthermore, sample data were collected from leaders of government agencies in Hainoi and Ho Chi Minh City in Vietnam. Results from such data may affect the generalizability of the findings. Future research should collect data from different cities in Vietnam and other countries such as China, India, Russia, etc., to validate the results in this study. In addition, several other variables may play important role in leaders’ decision-making about public health. For example, leaders’ characteristics (e.g., age, gender, education, etc.) and psychological factors (e.g., emotional intelligence, thinking styles, leadership styles, etc.) These variables should be investigated in future research.

## Figures and Tables

**Table 1:** Measurement items.

Variable	Item	Source
Leaders’ future orientation (LFO)	I spend time thinking about what our country’s future might be like	[13]
	I think a lot about what our country will be some day	
	Many of us tend to daydream about the future. It also happens to me	
	I often think about the things I am going to do in the future	
Self-efficacy (SEE)	I can always manage to solve difficult problems if I try hard enough.	[18]
	If someone opposes me, I can find means and ways to get what I want.	
	It is easy for me to stick to my aims and accomplish my goals.	
	I am confident that I could deal efficiently with unexpected events.	
	Thanks to my resourcefulness, I know how to handle unforeseen situations.	
	I can solve most problems if I invest the necessary effort.	
	I can remain calm when facing difficulties because I can rely on my coping abilities.	
	When I am confronted with a problem, I can usually find several solutions.	
	If I am in a bind, I can usually think of something to do.	
	No matter what comes my way, I am usually able to handle it.	
Perceived social support (PSS)	In a difficult situation, I can find help from my residents and colleagues.	[61]
	I can find emotional, informational and social support that I need from my residents and colleagues.	
	I can express my problems with my residents, colleagues and friends	
Public health investment intention (PHII)	We intend to invest more in public health programs.	[62]
	We will regularly invest in public health for our residents	
	We intend to continue investment in public health for our residents	

**Table 2:** Demographics of respondents.

Variable	Frequency	Percent
Gender		
Female	104	27.3%
Male	277	72.7%
Age		
30 or below	74	19.4%
31–40	223	58.5%
41–50	58	15.2%
51–60	26	6.8%
61 or above	0	0.0%
Marital status		
Married	277	72.7%
Not married	104	27.3%
Income		
Under 200 USD	126	33.1%
200-under 400 USD	224	58.8%
400-under 600 USD	24	6.3%
600-under 800 USD	6	1.6%
800 USD or above	1	0.3%
Education		
Undergraduate or below	260	68.2%
Master	116	30.4%
Ph.D.	5	1.3%
Tenure (years)		
Under 5 years	35	9.2%
5-under 10 years	173	45.4%
10 years or above	173	45.4%

Note: *n* = 381.

**Table 3:** Descriptive statistics and Pearson correlation.

Variable	Mean	SD	1	2	3	4
1. Leaders’ future orientation	3.65	0.84	0.86			
2. Self-efficacy	3.76	0.73	0.47 **	0.72		
3. Perceived social support	3.98	0.79	0.40 **	0.40 **	0.78	
4. Public health investment intention	3.62	0.87	0.47 **	0.48 **	0.41 **	0.84

Note: *n* = 381, ** *p* < 0.01, values of square roots of AVE are on the main diagonal.

**Table 4:** Confirmatory factor analysis results.

Constructs	Items	Loadings	CR	AVE	√AVE	Cronbach’s α
Leaders’ future orientation (LFO)	LFO1	0.86 ***	0.92	0.74	0.86	0.92
	LFO2	0.82 ***				
	LFO3	0.89 ***				
	LFO4	0.87 ***				
Self-efficacy (SEE)	SEE1	0.71 ***	0.91	0.52	0.72	0.91
	SEE2	0.67 ***				
	SEE3	0.73 ***				
	SEE4	0.72 ***				
	SEE5	0.72 ***				
	SEE6	0.71 ***				
	SEE7	0.74 ***				
	SEE8	0.72 ***				
	SEE9	0.78 ***				
	SEE10	0.69 ***				
Perceived social support (PSS)	PSS1	0.85 ***	0.83	0.62	0.78	0.83
	PSS2	0.79 ***				
	PSS3	0.71 ***				
Public health investment intention (PHII)	PHII1	0.84 ***	0.88	0.70	0.84	0.87
	PHII2	0.90 ***				
	PHII3	0.77 ***				

Note: *n* = 381, *** *p* < 0.001.
